# Complete Surgical Enucleation of a Giant Chylous Mesenteric Cyst

**DOI:** 10.1155/2020/4279345

**Published:** 2020-03-18

**Authors:** Maria Isaia, Maria Erodotou, Georgios Nakos, Nikolaos Nikolaou

**Affiliations:** ^1^General Surgery Department, Larnaca General Hospital, Larnaca, Cyprus; ^2^Pathology Department, Nicosia General Hospital, Nicosia, Cyprus; ^3^Medical School, University of Cyprus, Nicosia, Cyprus

## Abstract

Mesenteric cysts are rare benign abdominal tumors, and they can appear anywhere in the mesentery of the gastrointestinal tract, from the duodenum to the rectum. They are generally asymptomatic and may present as an incidental finding. The diagnosis is confirmed by the laparotomy findings and the results of the histopathological examination. Complete surgical (open or laparoscopic) enucleation of the cyst is the treatment of choice. We present a case of a female patient who presented with abdominal pain and a giant palpable abdominal mass. The patient underwent a surgical exploration which showed a giant mesenteric cyst. A complete surgical enucleation of the cyst was successfully performed without the need of bowel resection. The histopathological examination of the cyst was compatible with the diagnosis of chylous mesenteric cyst.

## 1. Introduction

Mesenteric cysts are rare benign pathologic entities with an incidence of 1 : 100.000 in adults and 1 : 20.000 in pediatric hospital admissions [1]. The pathogenesis remains unknown, and they can appear anywhere in the mesentery of the gastrointestinal tract, from the duodenum to the rectum. They are generally asymptomatic and may present as an incidental finding. The diagnosis is confirmed by the laparotomy findings and the results of the histopathological examination. Chylous cysts represent 7,3 to 9,9% of all abdominal cysts [2]. Only 50 cases had been reported up to 1987. A systematic literature review identified 19 cases of chylous mesenteric cysts in adults from 1980 to 2012 [3], while another review identified 16 cases from 2000 to 2018 [4].

## 2. Case Presentation

**Figure 1:**
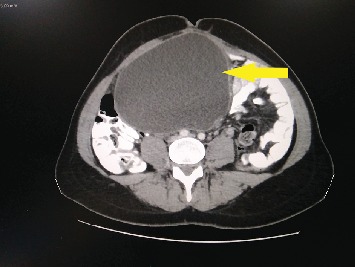
CT scan of the mesenteric cyst (arrow).

**Figure 2:**
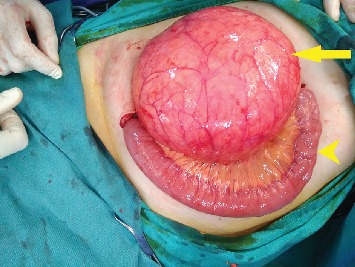
The mesenteric cyst (arrow) enveloped by the jejunum (pointed arrow).

**Figure 3:**
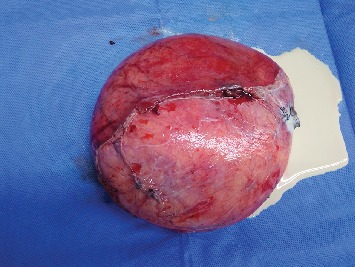
Surgical specimen of the cyst and its white milk-like content.

A 34-year-old woman presented with abdominal pain of 4-day duration. Her past medical history was clear, and a cesarean section was reported from her surgical history. On physical examination, a giant palpable mass was discovered on her right upper abdomen. Her vital signs were stable, and the laboratory tests were normal. An ultrasound of the abdomen showed a well-defined 15, 2 × 10 cm cystic mass, with a probable diagnosis of enteric duplication cyst. A CT scan of the abdomen was performed and revealed a giant cyst measuring 15, 9 × 14, 4 × 11, 2 cm with a thin wall and in contact with the right kidney and the uterus (Figure 1). The differential diagnosis included a mesenteric or an ovarian cyst. The gynecological examination failed to show any relation of the cyst with the ovaries. An exploratory laparotomy was decided. A giant mesenteric cyst was found at 60 cm distal to the ligament of Treitz, which was enveloped by 60 cm of the jejunum (Figure 2). The cyst was carefully dissected from the mesentery to spare the blood supply of the small bowel. Perforation of the cyst during the dissection revealed its content to be a dense white milk-like fluid (Figure 3). Successful enucleation was accomplished without the need of a bowel resection. The postoperative course was uneventful, and the patient was discharged on the third postoperative day.

**Figure 4:**
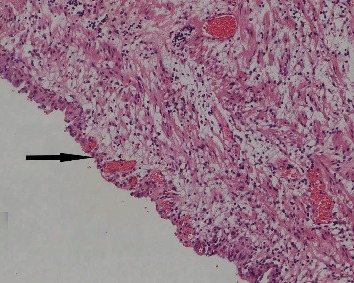
Cystic lesion is coated by mature mesothelial cells at a thickness of one to three cells (arrow). No signs of atypia (H-E ×100).

The histopathological examination showed a unilocular cyst with a smooth internal surface and a wall thickness 0,1–0,8 cm. The cyst wall was composed of vascular, connective, and fatty tissue with a small amount of smooth muscle fibers. Τhe stroma showed focal edema, focal mild lymphoplasmacytic infiltrates, and a few neutrophil polymorphonuclear cells. Focal lining of mesothelial type cells was recognized (Figure 4). These findings were compatible with a simple mesothelial cyst of the mesentery.

## 3. Discussion

Mesenteric cysts are rare benign pathologic entities with an incidence of 1 : 100.000 in adults and 1 : 20.000 in pediatric hospital admissions [1]. In 1507, Benevenni, an Italian anatomist, first illustrated this abdominal finding while performing an autopsy on an 8-year-old boy. In 1842, von Rokitansky was the first to announce the definition of a chylous mesenteric cyst, and in 1880, Tillaux achieved the first successful surgical excision of a cystic mass in the mesentery [5].

Mesenteric cysts may appear anywhere in the mesentery of the gastrointestinal tract, from the duodenum to the rectum. In a review of 162 cases by Kurtz et al., 60% of the mesenteric cysts occurred in the small bowel mesentery (most frequently in the ileum), 24% in the large bowel mesentery (usually in the ascending colon), 14,5% in the retroperitoneum, and 1,5% were undefined [6].

According to the histopathological findings, mesenteric cysts are classified in six groups: cysts of lymphatic origin (simple lymphatic cysts and lymphangiomas), mesothelial origin (simple mesothelial cysts, benign cystic mesotheliomas, and malignant cystic mesotheliomas), enteric origin (enteric duplication cysts and enteric cysts), urogenital origin, mature cystic teratomas (dermoid cysts), and nonpancreatic pseudocysts (traumatic and infectious origin) [7]. The findings of the present case were compatible with a simple mesothelial cyst.

Based on their etiology, Beahrs et al. classified mesenteric cysts in four groups: embryonic and developmental cysts (enteric, urogenital, lymphoid, and dermoid cysts), traumatic or acquired cysts, neoplastic cysts (benign and malignant cysts), and last infective and degenerative cysts (mycotic, parasitic, or tuberculous origin) [5]. The present case falls under the first group as indicated by the well-defined cyst wall and the presence of smooth muscle.

Mesenteric cysts can be single or multiple and unilocular or multilocular, and based on their content, they are described as serous, chylous, hemorrhagic, chylolymphatic, or infected [8, 9]. In the present case, the cyst was single and unilocular with a chylous content. Malignant transformation may occur in 3% of them. Serous cysts usually appear in the distal small bowel or colonic mesentery; chylous cysts appear in the proximal small bowel mesentery, as in the present case; and hemorrhagic cysts are caused by trauma and can appear anywhere in the bowel [10]. Chylous cysts represent 7,3 to 9,9% of all abdominal cysts [2].

Mesenteric cysts usually appear in the fifth decade, and they show female predominance [7]. They are generally asymptomatic and may present as an incidental finding. However, patients may report nonspecific symptoms, like pain (82%) and abdominal mass (61%), as in the present case, nausea and vomiting (45%), constipation (27%), and diarrhea (6%) [8]. The clinical manifestation will depend on the type, size, location, and complications of the cyst such as intestinal obstruction, volvulus, rupture, infection, peritonitis, shock, hemorrhage, and death. As a consequence, preoperative diagnosis is difficult. Ultrasound, CT scan, and MRI can show the size, the location, the relationship of the cyst to adjacent organs, the thickness of the wall, and the presence of fluid-fluid levels, as a result of an upper fluid level by chyle and a lower fluid level of the lymph [9, 10]. The diagnosis is confirmed by the laparotomy findings and the results of the histopathological examination [8]. Long pedicled mesenteric cysts may appear to originate from the ovaries, as in the case of a female patient with an initial diagnosis of ovarian cyst that was found to be a chylous mesenteric cyst during laparoscopy [3].

Many approaches have been described for the management of mesenteric cysts, such as marsupialization, sclerotherapy, drainage, enucleation, percutaneous aspiration, and en bloc resection of the cyst with the involved adjacent organs (bowel, gallbladder, pancreas, and spleen). Due to high recurrence and infection rates related to marsupialization, drainage, and aspiration, complete surgical (open or laparoscopic) enucleation of the cyst is the treatment of choice [9, 10]. In a similar case of a giant chylolymphatic mesenteric cyst [11], the authors emphasize the importance of successful enucleation of the cyst, irrespective of its size, due to its independent blood supply, as opposed to enterogenous cyst. A successful enucleation was accomplished in the present case without the need of bowel resection. In cases where the cyst involves blood vessels of the bowel, or complete enucleation cannot be achieved safely due to adhesions, bowel removal may be needed to succeed a complete resection of the cyst. It has been noted that bowel resection is required for one out of three of the treated patients [1]. In children, a bowel resection is frequently required [9]. Laparoscopic surgery is an alternative technique for cyst removal and has many advantages over open approach, including minimal trauma, less postoperative pain, shorter hospital stay, and earlier return to normal activity [12, 13].

## 4. Conclusion

Mesenteric cysts are rare benign abdominal tumors. Preoperative diagnosis is difficult due to their noncharacteristic clinical presentation. The diagnosis is proven after surgical exploration and the results of the histopathological findings. Complete surgical enucleation of the cyst, open or laparoscopic, is the gold standard approach for the management of a giant chylous cyst.
